# Lung cancer risk among workers in the construction industry: results from two case–control studies in Montreal

**DOI:** 10.1186/s12889-015-2237-9

**Published:** 2015-09-22

**Authors:** Aude Lacourt, Javier Pintos, Jérôme Lavoué, Lesley Richardson, Jack Siemiatycki

**Affiliations:** University of Montreal Hospital Research Center (CRCHUM), 850 rue St-Denis, Montreal, Qc H2X 0A9 Canada; Université de Bordeaux, ISPED, Centre INSERM U897-Epidemiologie-Biostatistique, F-33000 Bordeaux, France; INSERM, ISPED, Centre INSERM U897-Epidemiologie-Biostatistique, F-33000 Bordeaux, France; Department of Environmental and Occupational Health, School of Public Health, University of Montreal, Montreal, QC Canada; Department of Social and Preventive Medicine, School of Public Health, University of Montreal, Montreal, QC Canada; Guzzo-Cancer Research Society Chair in Environment and Cancer, School of Public Health, University of Montreal, Montreal, QC Canada

## Abstract

**Background:**

Given the large number of workers in the construction industry, it is important to derive accurate and valid estimates of cancer risk, and in particular lung cancer risk. In most previous studies, risks among construction workers were compared with general populations including blue and white collar workers. The main objectives of this study were to assess whether construction workers experience excess lung cancer risk, and whether exposure to selected construction industry exposures carries excess risks. We wished to address these objectives within the sub-population of blue collar workers.

**Methods:**

Two case-control studies were conducted in Montreal. Combined, they included 1593 lung cancer cases and 1427 controls, of whom 1304 cases and 1081 controls had been blue collar workers. Detailed lifetime job histories were obtained and translated by experts into histories of exposure to chemical agents. The two key analyses were to estimate odds ratio (OR) estimates of lung cancer risk: a) for all blue-collar construction workers compared with other blue-collar workers, and b) for construction workers exposed to each of 20 exposure agents found in the construction industry compared with construction workers unexposed to those agents. All analyses were conducted using unconditional logistic regression adjusted for socio-demographic factors and smoking history.

**Results:**

The OR for all construction workers combined was 1.11 (95 % CI: 0.90–1.38), based on 381 blue collar construction workers. Analyses of specific exposures were hampered by small numbers and imprecise estimates. While none of 20 occupational agents examined was significantly associated with lung cancer, the following agents manifested non-significantly elevated ORs: asbestos, silica, Portland cement, soil dust, calcium oxide and calcium sulfate.

**Conclusions:**

Compared with other blue collar workers, there was only a slight increased risk of lung cancer for subjects who ever held an occupation in the construction industry. The analyses of agents within the construction industry produced imprecise estimates of risk, but nevertheless pointed to some plausible associations. Excess risks for asbestos and silica were in line with previous knowledge. The possible excess risks with the other inorganic dusts require further corroboration.

**Electronic supplementary material:**

The online version of this article (doi:10.1186/s12889-015-2237-9) contains supplementary material, which is available to authorized users.

## Background

The construction industry remains one of the major sources of employment in industrialized countries. In Canada, according to the 2006 census, it is estimated that 10.4 % of male workers and 1.6 % of female workers were employed in this industry [1].

Construction workers can be grouped into “skilled” trades such as painters, carpenters, plumbers, electricians, roofers, and ironworkers, and unskilled construction “laborers” who perform a variety of job tasks in support of the former. Construction workers may be exposed to numerous physical and chemical agents like asbestos, silica, other dusts, solvents and other chemicals. Indeed, a construction worker is potentially exposed not only to materials used in his own trade but also to substances used by other trades in shared work environments. NIOSH listed approximately 70 different substances to which U.S. construction workers are potentially exposed [2].

Since 1990, several cohort studies conducted in different countries found significantly elevated mortality for lung cancer among construction workers [3–12] although some failed to find an excess risk [13–16]. Elevated mortality for lung cancer has also been reported for several specific construction trades such as bricklayers [4, 7, 10, 12, 16–21]; craftworkers [17, 19]; electricians [7, 20]; carpenters [4, 6, 7, 12, 18, 22, 23]; painters [4, 7, 12, 18, 24]; operating engineers [25]; roofers, waterproofers, allied workers [4, 10, 24]; and insulation workers [4, 6, 7, 15, 25].

Most retrospective cohort studies used the general population as the reference. This entails potential confounding biases related to several lifestyle or constitutional factors. The most important such factor, potentially, for lung cancer studies is smoking. Additionally, retrospective cohort studies of construction workers are limited by some characteristics of this industry: large numbers of relatively small employers, multi-employer work sites, and a highly mobile workforce. Case–control studies, where lifetime work histories can be elicited from study subjects, can overcome some of the limitations of cohort studies, but the comparisons between construction workers and the rest of the population remains vulnerable to confounding. A different paradigm would entail comparing risk of cancer among workers in the construction industry with risks among other blue-collar occupations.

Several exposures commonly found in the construction industry have been shown to carry excess risk of lung cancer, but many have not been adequately examined for carcinogenicity. Further, even for those shown to be carcinogenic, the evidence was often based on studies outside the construction industry or on studies that included non-construction as well as construction workers. Because of the unique characteristics of the workers and the work in this industry, we believe it is pertinent to assemble information on risks of lung cancer from the main agents in the construction industry, as they are experienced in that industry.

Given the large number of workers in the construction industry, it is important to derive accurate and valid estimates of cancer risk, and in particular lung cancer risk. The present article aims to provide evidence on two questions: 1) Do construction workers have an excess risk of lung cancer, when compared with other blue collar workers? 2) Which agents commonly found in the construction industry environment, carry an excess risk of lung cancer, under the conditions encountered by construction workers?

The data to address these objectives come from two population-based case control studies conducted in Montreal to explore possible associations between nearly 300 occupational substances and cancer. The first study included several sites of cancer including lung cancer [26], while the second study included only lung cancer [27]. Data from these studies included detailed information about occupational history, smoking behavior and many other personal characteristics.

## Methods

### Study population

Data for the present study come from two case–control studies conducted in the greater Montreal area. Details of subject ascertainment, data collection and exposure assessment were previously described [26–29].

The first study (Study I) was a population-based case–control study including 21 sites of cancers conducted between 1979 and 1986. Cases were histologically confirmed and were restricted to males aged between 35 and 70 years. Population controls were selected from electoral lists and frequency matched by age. The response rates among eligible subjects were 79 % among cases and 72 % among controls. From Study I, 857 male lung cancer cases and 533 male controls were available for these analyses.

The second study (Study II) was a population-based case–control study focused on lung cancer and included cases diagnosed between 1996 and 1998. Cases were histologically confirmed and included both males and females aged between 35 and 75 years. Controls were randomly sampled from population based electoral lists, stratified by sex and age to the distribution of cases. The response rates were 84 % among cases and 69 % among controls. From Study II, 736 cases and 894 controls were available for these analyses. Due to the small number of females in the construction industry, analyses were carried out among males only.

This research was approved by the ethics committees of the Institut national de recherche scientifique-IAF and McGill University. Ethics approval was also received from the following Montreal-area hospitals in which subjects were recruited: Montreal Chest Hospital Centre, Centre Hospitalier de Verdun, Cité de la santé, Hôpital Général Fleury, Hôtel Dieu de Montréal, Hôpital Jean-Talon, Hôpital Maisonneuve-Rosemont, Hôpital Notre Dame, Hôpital Sacré-Coeur, Hôpital St-Luc, Jewish General Hospital, Lakeshore Hospital, Montreal General Hospital, Queen Elizabeth Hospital, Reddy Memorial Hospital, Royal Victoria Hospital, Hôpital Santa Cabrini, Hôpital Ste Jeanne-d’Arc and St Mary’s Hospital. All participants provided informed consent.

### Data collection

A standardized questionnaire was administered by a trained interviewer to each subject or to a proxy respondent in case of death or if the subject was too ill to answer himself. The questionnaires were virtually identical in the two studies and entailed two parts: i) a structured section related to socio-demographic characteristics and lifestyle habits including detailed history of smoking; and ii) a semi-structured section related to employment history. For each job held for at least 6 months during the working lifetime history, information was solicited by means of probing questions on several characteristics such as company name, occupation title, general description of the work environment, main tasks performed, equipment and products used, use of protective equipment, and other information. To aid the interviewers to ask focused and technically appropriate questions, specialized questionnaires were developed for complicated occupations including brick and stone masons, concrete and terrazzo workers, carpenters, welders, braizers and solderers, plumbers and pipefitters, insulation workers, construction painters and cabinet makers [30, 31]. These were used whenever an interviewer was confronted with a subject who had been in one of these occupations.

Each recorded job was coded according to the Canadian Classification and Dictionary of Occupations of 1971 (CCDO) [32] and the Canadian Standard Industrial Classification (CSIC) of 1970 for study I [33] and 1980 for study II [34].

### Retrospective exposure assessment

A team of chemists and industrial hygienists examined each completed questionnaire and translated each job into a list of potential exposures using a checklist of 294 agents that included several substances of interest, such as crystalline silica, asbestos, asphalt, and diesel emissions.

Approximately 25,000 jobs were evaluated in both studies combined. The team of experts spent about 40 person-years on this project, including helping to develop the methodology, monitoring the quality of the interviewing, conducting background research on exposures in different occupations, coding the individual participants’ files, and recoding after the initial complete round of coding was finished. The final exposures attributed to a subject were based on consensus among the experts, who were blinded to the subject’s case or control status. For each substance considered present in each job, the coders noted three dimensions of information, each on a three-point scale: degree of confidence that the exposure had actually occurred (possible, likely, definite), average frequency of exposure per workweek (<5 %, 5 to 30 %, >30 % time of the week), and relative intensity of concentration of the agent (low, medium, high). Concentration levels were established with reference to benchmark occupations in which exposure to the substance is present. We identified certain workplace situations *a priori* which correspond to low, medium and high exposure for each substance, and the experts rated each reported job against these benchmarks. Non-exposure was interpreted as exposure up to the level that can be found in the general environment. When subjects self-reported that they had been exposed to some agent, the information was noted but not accepted at face value, unless our experts agreed that such exposures occurred. The exposure assessment was based not only on the worker’s occupation and industry, but also on individual characteristics of the workplace and tasks reported; an illustrative example is in the Appendix of Parent et al. [35]. Periodic tests showed a satisfactory degree of reproducibility among our experts and between our experts and others [36–38]. There was no evidence that cases provided more complete or more valid job histories than controls, as judged by the numbers of jobs reported per subject and by the interviewers’ rating of the quality of interviews. More detail can be found in Siemiatycki et al., 1991 [39].

### Statistical methods

First, we had to operationalize the designation of “construction worker” and we had to designate an appropriate comparison population. For ease of comparability and interpretation, a construction worker was defined as a subject who held at least one job in the construction industry (as it is referred to in the Canadian Standard Industrial Classifications of 1970 and 1980). In previous studies of construction workers (mostly cohort studies), the most commonly used comparison population had been the general population of the country in which the cohort was located. In designating a comparison population, there are several considerations. First, we would like the comparison population to be as similar as possible to the construction worker population, without the specific occupational conditions of construction workers. Second we would like to maximize the generalizability of the comparison. And third, we would like to maximize the statistical power of the comparison. These various *desiderata* lead to different choices and indeed we used two different strategies for a comparison population: i) the general population, using the entire case–control dataset (which enhances generalizability and power); and ii) non-construction blue-collar workers in the case–control dataset (which enhances comparability). The latter required an operational definition of “blue-collar worker”. We defined a blue-collar worker as one for whom over 50 % of the entire number of years of employment were spent in blue-collar occupations, as proposed by Ahrens et al. [40].

Odds ratios (ORs) were estimated by unconditional logistic regression models adjusted for age, ethnicity, socioeconomic status (SES) measured by the median family income for census tract of residence and by education level, and smoking history as synthesized by the comprehensive smoking index (CSI). The CSI integrates into a single index information on smoking duration, smoking intensity, and time since cessation and it has been shown to be useful for the purpose of adjusting for smoking in lung cancer studies [41]. We first performed analyses for each study separately, and then pooled the studies using models in which a binary variable for study was added to the covariates. To address the question of whether construction workers have an excess risk of lung cancer, subjects who held at least one occupation in the construction industry (as previously defined) were compared to those who never held an occupation in the sector. First we included all study subjects in an analysis, and second we restricted the study base to blue-collar workers in the study sample. These analyses were further stratified by large subdivision of the construction industry and by duration of employment in construction.

To examine which agents commonly found in the construction industry environment carry an excess risk of lung cancer, we restricted the study base to subjects who had ever worked in the construction industry. That is, for each agent, the reference category consisted of those construction workers not exposed to that agent.

We first established a list of agents to consider. Among the 294 agents in the database of agents coded by our exposure experts, we selected those that satisfied the following criteria: the lifetime prevalence of exposure to the agent among construction workers was more than twice that among non-construction workers, at least ten cases or ten controls were exposed among workers who had the Construction industry as the industry code, and the lifetime prevalence of exposure to the agent among controls was greater than 5 %. Twenty agents satisfied these criteria.

For each agent we derived an exposure index, based on the reliability of exposure, the frequency of exposure, the total duration of exposure and the relative concentration of exposure. The index has three categories: i) non-exposure; ii) non-substantial exposure; and iii) substantial exposure. Non-exposed is self-explanatory. Substantial exposure comprised subjects who had been exposed with a likely or definite reliability to medium or high concentrations for more than 5 % of their workweek and for at least 5 years [29]. Non-substantial exposure comprised those with less than substantial exposure.

Differences in ORs between the two studies were tested by introducing an interaction term between the binary indicator for study and the exposure variable of interest.

### Sensitivity analyses

Because of the relatively high number of proxy respondents, we conducted a whole set of analyses, parallel to the main ones, but restricted to subjects who responded for themselves.

There is an ongoing debate as to whether it is appropriate to adjust for markers of socioeconomic status (SES) in occupational studies [42–44], with some arguing that SES is a confounder to be adjusted and others that it is a collider to be omitted from statistical models. To examine whether inclusion of SES has the potential to bias the association between construction exposures and lung cancer, we conducted a sensitivity analysis in which we compared results from two models, one with adjustment for SES markers (income and education), and one without such adjustment. The other core covariates remained in both models.

To explore whether the selected cut-point of 50 % of working life in blue-collar occupations as a definition of a blue-collar worker influenced the results unduly, we also carried out entire sets of analyses using alternative cut-points, ranging from 25 to 75 %.

For the analyses of occupational chemical agents, we carried out the main set of analyses using as covariates various non-occupational covariates (age, socioeconomic status, education level, ethnicity and smoking history as synthesized by the CSI). In sensitivity analyses, we added to the models some other occupational exposure covariates that might confound the exposure-cancer associations. These covariates were chosen with two criteria in mind: i) classified by IARC as a Group 1 carcinogen for lung cancer [45] and ii) prevalence of exposure of at least 5 % in our study population. The agents thereby included were: asbestos, crystalline silica, and diesel engine emissions. The reason for not considering this as the main set of results is that it could obscure real associations if multiple agents, all measured with error, and probably with correlated error, are allowed to “soak up” some of the explanatory power of the agent being assessed. In this regard we prefer to avoid the possibility of over-adjustment, though we show the alternative results in Supplemental Digital Content Tables.

## Results

Selected characteristics of study subjects are presented in Table 1. Since the upper end of the eligible age range was slightly older in Study II than Study I, the mean age is slightly older in Study II. Subjects were mainly French Canadian and most subjects responded for themselves to the questionnaire. Between 20 and 30 % of subjects ever held an occupation in the construction industry, with slightly more cases than controls in both studies (24.2 % *vs* 21.2 % in study I and 28.1 % *vs* 23.3 % in study II). Among subjects who ever worked in construction, the mean duration of employment in the industry was approximately 16 years, whereas mean duration of employment in other industries was approximately 25 years. Only 11.3 % of workers who ever worked in construction had worked only in this industry throughout their lives.

**Table 1:** Distribution of main characteristics of subjects included in two case–control studies conducted in Montreal, QC, Canada

	Study I : 1979–1986		Study II : 1996–2002	
	Cases (857)	Controls (533)	Cases (736)	Controls (894)
Age (years), mean (SD^a^)	59.3 (7.0)	59.6 (7.9)	64.1 (7.9)	65.0 (7.6)
Ethnicity (%)				
French	592 (69.1)	342 (64.2)	570 (77.4)	576 (64.4)
British Isles	116 (13.5)	75 (14.1)	34 (4.6)	57 (6.4)
Other	149 (17.4)	116 (21.8)	132 (17.9)	261 (29.2)
Educational level (%)				
Primary	260 (30.3)	108 (20.3)	328 (44.6)	316 (35.3)
Secondary	489 (57.1)	299 (56.1)	315 (42.8)	372 (41.6)
Postsecondary	108 (12.6)	126 (23.6)	93 (12.6)	206 (23.0)
Respondent status (%)				
Self	605 (70.6)	466 (87.4)	443 (60.2)	807 (90.3)
Proxy	252 (29.4)	67 (12.6)	293 (39.8)	87 (9.7)
Median family income^b^ (in Can$), mean (SD)	22386.2 (7640.3)	26627.5 (8533.6)	32961.1 (14948.6)	35187.4 (14097.0)
Smoking status (%)				
Never	13 (1.5)	105 (19.7)	18 (2.4)	158 (17.7)
Former	159 (18.6)	178 (33.4)	205 (27.9)	461 (51.6)
Current^c^	685 (79.9)	250 (46.9)	513 (69.7)	275 (30.7)
Held a job in the construction industry (%)				
Never	650 (75.8)	420 (78.8)	529 (71.9)	686 (76.7)
Ever	207 (24.2)	113 (21.2)	207 (28.1)	208 (23.3)
Only in the construction industry	26 (3.0)	9 (1.7)	27 (3.7)	21 (2.3)
Both construction and other industries	181 (21.1)	104 (19.5)	180 (24.5)	187 (20.9)
Years worked in construction industry- mean (SD)	18.1 (12.8)	16.9(12.2)	17.9 (13.6)	19.3(14.8)

^a^ *SD* Standard Deviation

^b^Median family income for census tract

^c^Current smokers and subjects who quit <2 years before recruitment

Table 2 presents ORs for the association between employment in the construction industry and lung cancer, using two study bases, the entire population and blue-collar workers. Table 2 shows the pooled results from Study I and Study II. In Additional file 1: Table S1, we show results separately for the two studies. While there are some differences between point estimates from the two studies, none of the differences are statistically significant. As seen in Table 2, when comparing construction workers with all other workers, the OR was 1.15 (95 % CI 0.94–1.41), and when conducting the comparison among blue-collar workers, the OR was only slightly lower at 1.11 (95 % CI 0.90–1.38). There were borderline significant excess risks among workers in the building, industrial and heavy construction sector. There was no indication that workers with long-term employment in the industry had higher risks than those with shorter employment.

**Table 2:** Odds ratio between lung cancer and ever having been employed in the construction industry, the reference unexposed category being either all workers outside the construction industry or all blue collar workers outside the construction industry, stratified by duration and sector of the industry, in the pooled set of two studies conducted in Montreal, Canada

	All workers			Blue collar workers^a^		
	Ca/Co^b^ (1,593/1,427)	OR^c^	95 % CI^d^	Ca/Co (1,313/1,081)	OR	95 % CI
Never in the construction industry	1179/1106	1.00	-	932/793	1.00	-
Ever in the construction industry	414/321	1.15	0.94–1.41	381/288	1.11	0.90–1.38
Sector of the construction industry						
Building, industrial, heavy construction^e^	249/195	1.26	0.98–1.62	227/170	1.23	0.94–1.61
Trades contracting^f^	202/163	1.02	0.78–1.33	187/152	0.98	0.74–1.29
Duration in the construction industry						
≥ 10 years	268/206	1.13	0.89–1.44	250/193	1.08	0.84–1.39
≥ 20 years	173/138	1.10	0.82–1.46	161/130	1.05	0.78–1.41
≥ 30 years	100/81	1.11	0.77–1.60	95/76	1.08	0.74–1.58

^a^At least 50 % of the entire working lifetime spent in blue collar occupations (as defined by Ahrens et al., 1998 [40])

^b^Number of cases/number of controls

^c^Odds ratio adjusted for age, median family income for census tract, comprehensive smoking index, respondent status, education level and ethnicity and a binary indicator for studies

^d^95 % confidence interval

^e^Building, industrial, heavy construction: codes 40 (building, developing and general contracting industries), 41 (industrial and heavy construction industries from the Canadian Standard Industrial Classification of 1980 [34] and codes 404 (building construction), 406 (highway, bridge and street construction) and 409 (other constructions) from the Canadian Standard Industrial Classification of 1970 [33].

^f^Trade contracting industries: codes 42 (trade contracting industries), 44 (service industries incidental to construction from the Canadian Standard Industrial Classification of 1980 [34] and code 421 (special trade contractors) from the Canadian Standard Industrial Classification of 1970 [33].

Table 3 presents ORs for the association between lung cancer and each of the 20 construction-related chemical agents that satisfied our inclusion criteria. Given the lesser precision of estimates in this table compared to Table 2, we present ORs with only one decimal point. The analysis was restricted to construction workers, and the reference category for each agent was specific to that agent rather than being common to all agents. The table shows ORs for workers ever exposed and for workers substantially exposed. The following agents manifested ORs with lower 95 % confidence limits of 0.8 or greater at the substantial exposure level: soil dust (1.9; 95 % CI: 1.1–3.4), asbestos (1.9; 95 % CI: 0.8–4.6), crystalline silica (1.7; 95 % CI: 1.0–3.0), Portland cement (1.7; 95 % CI: 0.9–3.2), calcium oxide (known as lime) (2.0; 95 % CI: 1.0–4.2) and calcium sulfate (known as gypsum) (1.5; 95 % CI: 0.9–2.5).

**Table 3:** Odds ratios between lung cancer and selected chemical agents in analyses restricted to construction workers, in the pooled set of studies

Chemical agent^a^	Never exposed Ca/Co^c^	Ever Exposed			Substantially Exposed^b^		
		Ca/Co^d^	OR^e^	95 % CI^f^	Ca/Co	OR	95 % CI
Inorganic insulation dust	272/204	142/117	0.9	0.7–1.2	50/33	1.1	0.6–2.0
Soil dust	237/171	177/150	1.1	0.9–1.5	76/32	1.9	1.1–3.4
Asbestos	292/224	122/97	1.2	0.9–1.5	25/12	1.9	0.8–4.6
Crystalline silica	170/159	244/162	1.2	0.9–1.5	71/37	1.7	1.0–3.0
Portland cement	283/215	131/106	1.1	0.8–1.4	52/27	1.7	0.9–3.2
Glass fibers	346/276	68/45	1.1	0.8–1.5	14/10	1.0	0.4–2.8
Brick dust	347/268	67/53	1.1	0.8–1.6	18/9	1.4	0.5–3.6
Concrete dust	235/155	179/166	0.9	0.7–1.2	69/48	0.9	0.5–1.5
Mineral wool fibers	298/233	116/88	1.1	0.8–1.4	29/21	1.2	0.5–2.5
Calcium oxide	352/277	62/44	1.1	0.8–1.6	36/18	2.0	1.0–4.2
Calcium sulfate	227/187	187/134	1.2	0.9–1.5	76/44	1.5	0.9–2.5
Calcium carbonate	320/230	94/91	1.0	0.8–1.4	27/19	1.2	0.6–2.6
Wood dust	204/141	210/180	0.9	0.7–1.1	86/58	0.8	0.5–1.3
Hydrogen chloride	352/256	62/65	0.8	0.6–1.1	10/10	0.6	0.2–1.6
Soldering fumes	364/271	50/50	0.9	0.6–1.2	18/18	0.7	0.3–1.5
Liquid fuel combustion products	369/279	45/42	0.9	0.6–1.3	12/19	0.4	0.2–1.0
Propane combustion products	372/288	42/33	1.3	0.8–1.9	8/8	1.1	0.3–3.7
Turpentine	381/301	33/20	1.2	0.8–2.0	17/11	1.1	0.4–2.9
Asphalt	374/277	40/44	0.7	0.5–1.1	14/16	0.4	0.2–1.0
Coal tar and pitch	387/297	27/24	0.8	0.5–1.4	12/5	1.4	0.4–5.2

^a^Criteria for selection of chemical agents: at least ten cases or ten controls with substantial exposure in the pooled studies; prevalence of exposure greater than 5 % among construction workers; and prevalence among controls at least twice as high in construction workers as in other workers

^b^Substantial exposure comprised subjects who had been exposed with a probable or definite reliability to medium or high concentrations for more than 5 % of their workweek and for at least 5 years

^c^For each chemical agent, the reference category consists of construction workers who were never exposed to the agent

^d^Number of cases/number of controls

^e^ *OR* Odds ratio adjusted for age, median family income for census tract, comprehensive smoking index, respondent status, education level and ethnicity and binary indicator for study

^f^95 % confidence interval

### Sensitivity analyses

A complete set of parallel analyses was conducted among self-respondents, i.e., excluding proxy respondents. Additional file 1: Table S2 is analogous to Table 2, but restricted to self-respondents. As expected, compared with the results among all respondents, those restricted to self-respondents entail wider confidence intervals. There was some indication that ORs were higher for construction workers in the analyses restricted to self-respondents, but little indication that ORs for specific exposures differed between the analyses among all respondents and those among self-respondents.

Some analyses were restricted to blue-collar workers. In order to assess whether the OR estimates in Table 2 were sensitive to the particular cut-point of 50 % used to define blue-collar workers, we also implemented analogous analyses using cut-points ranging from 25 to 75 %. Additional file 1: Table S3 shows results analogous to those shown in Table 2, but using 25 and 75 % as cut-points, as well as 50 %. The OR estimate for ever employed in the construction industry among blue-collar workers varied from 1.16 to 1.13 as we varied the blue-collar career cut-point across the range from 25 to 75 %; in other words it had little impact.

Additional file 1: Table S4 is analogous to Table 3, but it contrasts the results using non-occupational confounder covariates with those using both non-occupational and occupational ones. Except for some slight variations, inclusion of the three occupational covariates did not materially change the ORs.

Additional file 1: Table S5 is analogous to Additional file 1: Table S4, but it is restricted to self-respondents. It shows that, apart from decreased precision of the estimates, the restriction to self-respondents has no important impact.

Additional file 1: Tables S6 and S7 are analogous to Tables 2 and 3 and show results of a sensitivity analysis in which we excluded and included two SES variables (median family income of the census tract and education level) from the statistical models. There was not really much impact of inclusion or exclusion of these covariates on the ORs between the various construction exposures and lung cancer. Hardly any of the OR pairings differed by more than one decimal point.

Table 4 shows the results of an analysis in which we explored potential effect modification by smoking for selected occupational agents. Namely, we selected those agents that, in Table 3, showed an OR ≥1.5 and sufficient numbers to support stratified analyses. Six agents were selected. Since there were very few never smokers among cases, the non-smokers category was supplemented with lifetime low intensity smokers. Operationally, we defined lifetime low intensity smokers as individuals having a CSI value below the 25th percentile on this scale. Smokers with CSI values above the 25th percentile were considered medium/heavy smokers. To evaluate if the ORs between occupational exposures and lung cancer differed between the two strata of smokers, we carried out an analysis based on all subjects including the two variables, smoking status and exposure to occupational chemical agent, by testing their cross-product term. The continuous CSI variable was maintained as a covariate in the models to avoid any residual confounding within the smoking status strata. There was no evidence of interaction in Table 4 except for results on silica. It appeared that there was a stronger effect of silica on lung cancer among non and low smokers than among medium-heavy smokers.

**Table 4:** Odds ratios between lung cancer and selected chemical agents in analyses restricted to construction workers, in the pooled set of studies, stratified by smoking status, and test for interaction

Chemical agent^a^	Smoking status								*P*-value^g^
	Never-low smokers (*n* = 229)				Medium-heavy smokers (*n* = 506)				
	Never exposed^c^	Ca/Co^d^	OR^e^	95 % CI^f^	Never exposed^c^	Ca/Co	OR^e^	95 % CI	
Soil dust									
*Ever exposed*	41/80	23/85	1.1	0.6–2.1	196/91	154/65	1.1	0.8–1.5	0,9687
*Substantially exposed* ^b^		13/18	2.9	1.0–8.9		63/14	1.8	0.9–3.6	0,8496
Asbestos									
*Ever exposed*	44/113	20/52	1.2	0.7–2.2	248/111	102/45	1.1	0.8–1.6	0,6778
*Substantially exposed*		5/5	2.3	0.4–14.5		20/7	1.8	0.7–4.9	0,7166
Crystalline silica									
*Ever exposed*	29/80	35/85	1.0	0.6–1.7	141/79	209/77	1.3	0.9–1.7	0,2623
*Substantially exposed*		17/18	3.1	1.0–9.6		54/19	1.4	0.7–2.7	0,0223
Portland cement									
*Ever exposed*	42/110	22/55	1.4	0.8–2.5	241/105	109/51	1.0	0.7–1.4	0,3067
*Substantially exposed*		13/14	4.0	1.2–13.4		39/13	1.3	0.6–2.8	0,4819
Calcium oxide									
*Ever exposed*	52/147	12/18	1.8	0.8–3.7	300/130	50/26	1.0	0.6–1.4	0,1193
*Substantially exposed*		9/10	3.2	0.9–12.0		27/8	1.7	0.7–4.1	0,5410
Calcium sulfate									
*Ever exposed*	36/95	28/70	1.0	0.6–1.8	191/92	159/64	1.2	0.9–1.6	0,8699
*Substantially exposed*		14/22	1.3	0.5–3.6		62/22	1.6	0.9–2.9	0,9670

^a^Criteria for selection of chemical agents: at least ten cases or ten controls with substantial exposure in the pooled studies; prevalence of exposure greater than 5 % among construction workers; and prevalence among controls at least twice as high in construction workers as in other workers

^b^Substantial exposure comprised subjects who had been exposed with a probable or definite reliability to medium or high concentrations for more than 5 % of their workweek and for at least 5 years

^c^For each chemical agent, the reference category consists of construction workers who were never exposed to the agent

^d^Number of cases/number of controls substantially exposed

^e^ *OR* Odds ratio adjusted for age, median family income for census tract, comprehensive smoking index, respondent status, education level and ethnicity and binary indicator for study

^f^95 % confidence interval

^g^Significance of the interaction term between smoking (binary) and occupational exposure (1) ever *vs* never exposed or 2) not exposed/not substantially exposed/substantially exposed, in the regression models

## Discussion

While there has been considerable research on cancer risks among construction workers as a whole as well as among some construction industry occupations (e.g., painters, welders) and on exposure agents found in this industry (e.g., asbestos, silica, wood dust), most of this research has used the retrospective cohort design with its attendant problems (difficult to control for smoking and other potential confounders, difficult to ascertain lifetime occupation histories, especially in an industry with frequent mobility between multiple small-scale employers). Further, insofar as research on agents is concerned, such research has usually been conducted in studies that have cut across all industries or that have been localized in industries other than construction. The same exposures may entail different risk profiles in different industries, because of differing covariates or differing exposure circumstances. Because of the large number of workers employed in the construction industry and its unique character, it is pertinent to address the issue of risks of lung cancer within this industry, among workers with different occupations and exposures in this industry.

Several cohort studies of construction workers as a whole reported elevated mortality or incidence for lung cancer with relative risk estimates in the range of 1.5 to 5.5 [3–12]. However, some other studies found no excess risk [13–16]. Several case–control studies also reported significant associations between construction industry and lung cancer risk [46–51], though some case–control studies had results closer to the null [52–56].

Comparing lung cancer risks in all construction workers combined with those in the general population, we found a slight borderline non-significant excess risk (OR = 1.15; 95 % CI: 0.9–1.4). There was some indication of higher risk among workers in the heavy construction sector, but no indication that long duration workers experienced higher risk. This latter finding may be due to a type of healthy worker effect if workers who were particularly susceptible to lung cancer or other respiratory diseases quit construction industry and left for other industries.

We also compared blue-collar construction workers to blue-collar workers in other industries. Whereas the analysis in the entire population simulates the paradigm of a cohort study in which the disease occurrence of the cohort of construction workers is compared with that of the general population, the analysis restricted to blue-collar workers represents the situation, difficult to achieve in a cohort study, where we lessen the opportunity for confounding by blue-collar/white collar status and its correlates. The resulting ORs were very similar to those using the entire study population as reference group. Nor was this finding sensitive to the particular cut-point we used to define a worker as blue-collar or not. Results based on self-respondents were similar to the main findings and adjustment for different combinations of covariates had little impact.

Some previous studies indicated stronger associations between work in construction industry and lung cancer than what we found. There may be more mobility between work in this industry and others in our study area than elsewhere, as evidenced by the fact that only 11 % of construction industry workers had never worked in any other industry, and on average, workers who had ever worked in this industry, spent less than half of their careers in this industry. Still, it is not implausible that workers in this industry as a whole experience only a slight (11 %) excess risk of lung cancer.

Our analyses of exposure agents (Table 3) were restricted to construction workers and therefore entailed rather small numbers and imprecise risk estimates. Whether to conduct such an analysis in the entire population or to restrict the study base to construction workers is not self-evident; each strategy presents advantages and disadvantages. Conducting the analysis in the entire population is the most common approach; it maximizes numbers of subjects and allows for information across the industrial spectrum to contribute to the risk estimates. However it leaves open the opportunity for residual confounding by covariates that are included in the analysis but measured with error, and by unmeasured confounders. Restricting these analyses to the sub-population of construction workers lessens the opportunity for such confounding. However, restriction may be criticized on the grounds that it entailed substantial loss of information or that it constitutes a form of overmatching. The latter assertion would be justifiable if construction workers constituted a relatively homogeneously exposed population. But that is not the case; the construction industry contains workers who have distinctive and different jobs and exposures, although some exposures cut across many occupations in the industry.

To the extent that there may be a general pervasive excess risk of lung cancer among construction industry workers, the intra-industry estimates we show for the agents in Table 3 may underestimate the true risks, but only slightly. Among the 20 agents we considered, most showed point estimates above 1.0, and some showed point estimates as high as or higher than 1.5. Given the relatively small numbers of subjects exposed to these agents within the industry, the confidence intervals were wide and they all included 1.0, except for soil dust. The most suggestively elevated ORs were for asbestos, crystalline silica, soil dust (mainly from excavation activities), Portland cement, calcium oxide (also known as lime) and calcium sulfate (mainly in the form of gypsum). Of these, of course asbestos and silica are known to be related to lung cancer [57] and in our own studies, when including data from across the industrial spectrum, the ORs for these agents were significantly elevated [58, 59]. There were no interactions between cigarette smoking and any of the occupational agents on lung cancer risk, except for silica; we found that the effect of exposure to silica was stronger among non and low smokers than among medium-heavy smokers. Synthesizing previous evidence, a recent meta-analysis did not find an increased risk of lung cancer associated with exposure to Portland cement (SMR = 1.1; [95 % CI:0.7–1.6]) [60]. We failed to find any study examining lung cancer risks in relation to calcium oxide, calcium sulfate or soil dust.

Given the lack of strong evidence of excess risks for any but the already recognized lung carcinogens, it is not particularly worrying that we did not control for mutual confounding by any but the already known carcinogens. The additional adjustment for asbestos, silica and diesel engine emissions did not have much impact on point estimates of risk for the other agents under study.

We included in the final models all variables that were considered as known or *a priori* confounders. A sensitivity analysis evaluating the impact of inclusion or exclusion of SES indicated that in this study, inclusion of SES, after including smoking and other *a priori* confounders, did not affect the results greatly. In themselves, these sensitivity analyses do not answer the question as to whether SES is a confounder or collider.

Occupational exposure was retrospectively assessed by industrial hygienists and chemists using a standardized method which has been shown to be reliable and valid [38, 61, 62], based on occupation histories obtained by trained interviewers via a method shown to be valid and reliable [63, 64]. Still such a method certainly entails measurement error, which should be non-differential with respect to case/control status since experts were blind to the disease status when they assessed each occupational history.

## Conclusion

In conclusion, our study suggests only a slight increased risk of lung cancer for subjects who ever held an occupation in the construction industry. In the analyses of agents within the construction industry, there were suggestions of increased risks among workers exposed to soil dust, asbestos, crystalline silica, Portland cement, calcium oxide, and calcium sulfate. For asbestos and silica exposure in the construction industry, this provides stronger evidence than previous studies which included many industries; for the other agents, it signals the need for corroborative evidence.

## Additional file

Additional file 1: Table S1. Odds ratios of lung cancer and ever being employed in the construction industry, the reference unexposed category being either all workers outside the construction industry or all blue collar workers outside the construction industry, in Study I and Study II, separately. **Table S2.** Odds ratios of lung cancer and ever being employed in the construction industry, the reference unexposed category being either all workers outside the construction industry or all blue collar workers outside the construction industry, pooled set of two studies conducted in Montreal, Canada, restricted to self-respondents. **Table S3.** Odds ratios of lung cancer and ever being employed in the construction industry, the reference unexposed category being all blue collar workers outside the construction industry, pooled set of two studies, with three different criteria for defining a blue collar worker*. **Table S4.** Odds ratios of lung cancer and exposure to selected chemical agents in analyses restricted to construction workers, pooled set of studies, using two sets of covariates. **Table S5.** Odds ratios of lung cancer and exposure to selected chemical agents in analyses restricted to construction workers, pooled set of studies, using two sets of covariates, and restricted to self-respondents. **Table S6.** Odds ratios of lung cancer and ever being employed in the construction industry, the reference unexposed category being either all workers outside the construction industry or all blue collar workers outside the construction industry, with or without adjustment for socio-economic status (SES, represented by median family income for census tract and education level). **Table S7.** Odds ratios of lung cancer and exposure to selected chemical agents in analyses restricted to construction workers, pooled set of studies, with or without adjustment for socio-economic status (SES, represented by median family income for census tract and education level). (DOCX 81 kb)

## Abbreviations

CCDO: Canadian Classification and Dictionary of Occupations
CI: Confidence Interval
CSI: Comprehensive smoking index
IARC: International Agency for Research on Cancer
NIOSH: The National Institute for Occupational Safety and Health
OR: Odds ratio
CSIC: Canadian Standard Industrial Classification
SMR: Standardized Mortality Ratio
US: United States
