# Supplementary material for: Lung cancer risk among workers in the construction industry: results from two case–control studies in Montreal
Source: BMC Public Health. 2015 Sep 22;15:941. doi: 10.1186/s12889-015-2237-9 (PMC4580354; doi:10.1186/s12889-015-2237-9)
Supplement: Additional file 1: Table S1. — Odds ratios of lung cancer and ever being employed in the construction industry, the reference unexposed category being either all workers outside the construction industry or all blue collar workers outside the construction industry, in Study I and Study II, separately. Table S2. Odds ratios of lung cancer and ever being employed in the construction industry, the reference unexposed category being either all workers outside the construction industry or all blue collar workers outside the construction industry, pooled set of two studies conducted in Montreal, Canada, restricted to self-respondents. Table S3. Odds ratios of lung cancer and ever being employed in the construction industry, the reference unexposed category being all blue collar workers outside the construction industry, pooled set of two studies, with three different criteria for defining a blue collar worker*. Table S4. Odds ratios of lung cancer and exposure to selected chemical agents in analyses restricted to construction workers, pooled set of studies, using two sets of covariates. Table S5. Odds ratios of lung cancer and exposure to selected chemical agents in analyses restricted to construction workers, pooled set of studies, using two sets of covariates, and restricted to self-respondents. Table S6. Odds ratios of lung cancer and ever being employed in the construction industry, the reference unexposed category being either all workers outside the construction industry or all blue collar workers outside the construction industry, with or without adjustment for socio-economic status (SES, represented by median family income for census tract and education level). Table S7. Odds ratios of lung cancer and exposure to selected chemical agents in analyses restricted to construction workers, pooled set of studies, with or without adjustment for socio-economic status (SES, represented by median family income for census tract and education level). (DOCX 81 kb) [file 12889_2015_2237_MOESM1_ESM.docx]

**Table S1**. Odds ratios of lung cancer and ever being employed in the construction industry, the reference unexposed category being either all workers outside the construction industry or all blue collar workers outside the construction industry, in Study I and Study II separately

|  | All workers | | | | | | |  | Blue collar workers* | | | | | | |
| --- | --- | --- | --- | --- | --- | --- | --- | --- | --- | --- | --- | --- | --- | --- | --- |
|  | Study I | | |  | Study II | | |  | Study I | | |  | Study II | | |
|  | Ca/Co^†^  (857/533) | OR^‡^ | 95% CI^§^ |  | Ca/Co  (736/894) | OR | 95% CI |  | Ca/Co  (703/397) | OR | 95% CI |  | Ca/Co  (610/684) | OR | 95% CI |
| Never in the construction industry | 650/420 | 1.00 |  |  | 529/686 | 1.00 |  |  | 510/294 | 1.00 |  |  | 422/499 | 1.00 |  |
| Ever in the construction industry | 207/113 | 0.95 | 0.70-1.30 |  | 207/208 | 1.25 | 0.95-1.65 |  | 193/103 | 0.93 | 0.67-1.29 |  | 188/185 | 1.20 | 0.90-1.62 |
| Sector of the construction industry |  |  |  |  |  |  |  |  |  |  |  |  |  |  |  |
| Building, industrial, heavy construction^\|\|^ | 116/58 | 1.02 | 0.68-1.52 |  | 133/137 | 1.38 | 1.00-1.91 |  | 109/51 | 1.03 | 0.68-1.58 |  | 118/119 | 1.30 | 0.92-1.85 |
| Trades contracting^¶^ | 112/67 | 0.90 | 0.61-1.32 |  | 90/96 | 1.07 | 0.73-1.55 |  | 102/64 | 0.82 | 0.55-1.23 |  | 85/88 | 1.07 | 0.72-1.59 |
| Duration in the construction industry |  |  |  |  |  |  |  |  |  |  |  |  |  |  |  |
| ≥ 10 years | 137/69 | 0.97 | 0.67-1.40 |  | 131/137 | 1.19 | 0.86-1.65 |  | 127/65 | 0.89 | 0.60-1.31 |  | 123/128 | 1.17 | 0.83-1.64 |
| ≥ 20 years | 87/48 | 0.97 | 0.63-1.50 |  | 86/90 | 1.15 | 0.78-1.71 |  | 81/46 | 0.90 | 0.57-1.41 |  | 80/84 | 1.13 | 0.75-1.70 |
| ≥ 30 years | 50/22 | 1.29 | 0.71-2.33 |  | 50/59 | 1.00 | 0.63-1.61 |  | 47/21 | 1.21 | 0.65-2.23 |  | 48/55 | 1.00 | 0.61-1.62 |

^*^ At least 50% of the entire working lifetime spent in blue collar occupations (as defined by Ahrens et al., 1998^40^)

^†^ Number of cases/number of controls.

^‡^ Odds ratio adjusted for age, median family income for census tract, comprehensive smoking index, respondent status, education level and ethnicity.

^§^ 95% confidence interval.

^||^ Building, industrial, heavy construction: codes 40 (building, developing and general contracting industries), 41 (industrial and heavy construction industries from the Canadian Standard Industrial Classification of 1980^34^ and codes 404 (building construction), 406 (highway, bridge and street construction) and 409 (other constructions) from the Canadian Standard Industrial Classification of 1970.^33^

^¶^ Trade contracting industries: codes 42 (trade contracting industries), 44 (service industries incidental to construction from the Canadian Standard Industrial Classification of 1980^34^ and code 421 (special trade contractors) from the Canadian Standard Industrial Classification of 1970.^33^

**Table S2**. Odds ratios of lung cancer and ever being employed in the construction industry, the reference unexposed category being either all workers outside the construction industry or all blue collar workers outside the construction industry, pooled set of two studies conducted in Montreal, Canada, restricted to self-respondents

|  | All workers | | |  | Blue collar workers* | | |
| --- | --- | --- | --- | --- | --- | --- | --- |
|  | Ca/Co^†^  (1,048/1,273) | OR^‡^ | 95% CI^§^ |  | Ca/Co  (864/957) | OR | 95% CI |
| Never in the construction industry | 745/985 | 1.00 |  |  | 585/696 | 1.00 |  |
| Ever in the construction industry | 303/288 | 1.20 | 0.96-1.50 |  | 279/261 | 1.13 | 0.89-1.44 |
| Sector of the construction industry |  |  |  |  |  |  |  |
| Building, industrial, heavy construction^\|\|^ | 182/178 | 1.28 | 0.98-1.69 |  | 165/158 | 1.20 | 0.89-1.60 |
| Trades contracting^¶^ | 152/146 | 1.08 | 0.81-1.44 |  | 141/136 | 1.02 | 0.75-1.39 |
| Duration in the construction industry |  |  |  |  |  |  |  |
| ≥ 10 years | 188/184 | 1.20 | 0.92-1.56 |  | 177/174 | 1.13 | 0.85-1.49 |
| ≥ 20 years | 123/125 | 1.12 | 0.82-1.54 |  | 116/120 | 1.05 | 0.75-1.45 |
| ≥ 30 years | 74/73 | 1.18 | 0.80-1.75 |  | 71/70 | 1.11 | 0.74-1.67 |

^*^ At least 50% of the entire working lifetime spent in blue collar occupations (as defined by Ahrens et al., 1998^40^)

^†^ Number of cases/number of controls.

^‡^ Odds ratio adjusted for age, median family income for census tract, comprehensive smoking index, respondent status, education level and ethnicity and a binary indicator for studies.

^§^ 95% confidence interval.

^||^ Building, industrial, heavy construction: codes 40 (building, developing and general contracting industries), 41 (industrial and heavy construction industries from the Canadian Standard Industrial Classification of 1980^34^ and codes 404 (building construction), 406 (highway, bridge and street construction) and 409 (other constructions) from the Canadian Standard Industrial Classification of 1970.^33^

^¶^ Trade contracting industries: codes 42 (trade contracting industries), 44 (service industries incidental to construction from the Canadian Standard Industrial Classification of 1980^34^ and code 421 (special trade contractors) from the Canadian Standard Industrial Classification of 1970.^33^

**Table S3.** Odds ratios of lung cancer and ever being employed in the construction industry, the reference unexposed category being all blue collar workers outside the construction industry, pooled set of two studies, with three different criteria for defining a blue collar worker*

|  | 25% of the career as blue collar worker | | |  | 50% of the career as blue collar worker | | |  | 75% of the career as blue collar worker | | |
| --- | --- | --- | --- | --- | --- | --- | --- | --- | --- | --- | --- |
|  | Ca/Co^†^  (1,402/1,179) | OR^‡^ | 95% CI^§^ |  | Ca/Co  (1,313/1,081) | OR | 95% CI |  | Ca/Co  (1,202/974) | OR | 95% CI |
| Never in the construction industry | 1000/878 | 1.00 |  |  | 932/793 | 1.00 | - |  | 837/701 | 1.00 |  |
| Ever in the construction industry | 402/301 | 1.16 | 0.94-1.44 |  | 381/288 | 1.11 | 0.90-1.38 |  | 365/273 | 1.13 | 0.90-1.41 |
| Sector of the construction industry |  |  |  |  |  |  |  |  |  |  |  |
| Building, industrial, heavy construction^\|\|^ | 239/180 | 1.27 | 0.98-1.64 |  | 227/170 | 1.23 | 0.94-1.61 |  | 218/158 | 1.31 | 0.99-1.73 |
| Trades contracting^¶^ | 199/155 | 1.05 | 0.80-1.38 |  | 187/152 | 0.98 | 0.74-1.29 |  | 177/146 | 0.93 | 0.70-1.24 |
| Duration in the construction industry |  |  |  |  |  |  |  |  |  |  |  |
| ≥ 10 years | 261/197 | 1.15 | 0.89-1.47 |  | 250/193 | 1.08 | 0.84-1.39 |  | 243/186 | 1.10 | 0.85-1.43 |
| ≥ 20 years | 167/131 | 1.12 | 0.83-1.50 |  | 161/130 | 1.05 | 0.78-1.41 |  | 158/126 | 1.07 | 0.79-1.45 |
| ≥ 30 years | 98/76 | 1.14 | 0.79-1.66 |  | 95/76 | 1.08 | 0.74-1.58 |  | 93/75 | 1.10 | 0.75-1.62 |

^*^ At least 25%, 50% or 75% of the entire working lifetime spent in blue collar occupations (as defined by Ahrens et al., 1998^40^)

^†^ Number of cases/number of controls.

^‡^ Odds ratio adjusted for age, median family income for census tract, comprehensive smoking index, respondent status, education level and ethnicity and a binary indicator for studies.

^§^ 95% confidence interval.

^||^ Building, industrial, heavy construction: codes 40 (building, developing and general contracting industries), 41 (industrial and heavy construction industries from the Canadian Standard Industrial Classification of 1980^34^ and codes 404 (building construction), 406 (highway, bridge and street construction) and 409 (other constructions) from the Canadian Standard Industrial Classification of 1970.^33^

^¶^ Trade contracting industries: codes 42 (trade contracting industries), 44 (service industries incidental to construction from the Canadian Standard Industrial Classification of 1980^34^ and code 421 (special trade contractors) from the Canadian Standard Industrial Classification of 1970.^33^

**Table S4**: Odds ratios of lung cancer and exposure to selected chemical agents in analyses restricted to construction workers, pooled set of studies, using two sets of covariates

| Chemical agent* | Never exposed^‡^ | Ever Exposed | | | | | |  | Substantially exposed^†^ | | | | | |
| --- | --- | --- | --- | --- | --- | --- | --- | --- | --- | --- | --- | --- | --- | --- |
|  |  | Ca/Co^§^ | OR_1_^\|\|^ | 95% CI^¶^ |  | OR_2_^**^ | 95% CI |  | Ca/Co | OR_1_ | 95% CI |  | OR_2_ | 95% CI |
| Inorganic insulation dust | 272/204 | 142/117 | 0.9 | 0.7-1.2 |  | 0.9 | 0.7-1.2 |  | 50/33 | 1.1 | 0.6-2.0 |  | 1.0 | 0.5-1.8 |
| Soil dust | 237/171 | 177/150 | 1.1 | 0.9-1.5 |  | 1.0 | 0.8-1.3 |  | 76/32 | 1.9 | 1.1-3.4 |  | 1.6 | 0.9-2.9 |
| Asbestos | 292/224 | 122/97 | 1.2 | 0.9-1.5 |  | 1.1 | 0.9-1.5 |  | 25/12 | 1.9 | 0.8-4.6 |  | 1.8 | 0.7-4.4 |
| Crystalline silica | 170/159 | 244/162 | 1.2 | 0.9-1.5 |  | 1.1 | 0.9-1.5 |  | 71/37 | 1.7 | 1.0-3.0 |  | 1.6 | 0.9-2.9 |
| Portland cement | 283/215 | 131/106 | 1.1 | 0.8-1.4 |  | 1.0 | 0.7-1.3 |  | 52/27 | 1.7 | 0.9-3.2 |  | 1.5 | 0.8-2.9 |
| Glass fibers | 346/276 | 68/45 | 1.1 | 0.8-1.5 |  | 1.1 | 0.7-1.5 |  | 14/10 | 1.0 | 0.4-2.8 |  | 1.0 | 0.4-2.8 |
| Brick dust | 347/268 | 67/53 | 1.1 | 0.8-1.6 |  | 1.0 | 0.7-1.5 |  | 18/9 | 1.4 | 0.5-3.6 |  | 1.2 | 0.5-3.2 |
| Concrete dust | 235/155 | 179/166 | 0.9 | 0.7-1.2 |  | 0.8 | 0.6-1.1 |  | 69/48 | 0.9 | 0.5-1.5 |  | 0.7 | 0.4-1.3 |
| Mineral wool fibers | 298/233 | 116/88 | 1.1 | 0.8-1.4 |  | 1.0 | 0.8-1.4 |  | 29/21 | 1.2 | 0.5-2.5 |  | 1.1 | 0.5-2.4 |
| Calcium oxide | 352/277 | 62/44 | 1.1 | 0.8-1.6 |  | 1.0 | 0.7-1.5 |  | 36/18 | 2.0 | 1.0-4.2 |  | 1.8 | 0.8-3.7 |
| Calcium sulfate | 227/187 | 187/134 | 1.2 | 0.9-1.5 |  | 1.2 | 0.9-1.5 |  | 76/44 | 1.5 | 0.9-2.5 |  | 1.5 | 0.9-2.5 |
| Calcium carbonate | 320/230 | 94/91 | 1.0 | 0.8-1.4 |  | 0.9 | 0.7-1.3 |  | 27/19 | 1.2 | 0.6-2.6 |  | 1.0 | 0.5-2.2 |
| Wood dust | 204/141 | 210/180 | 0.9 | 0.7-1.1 |  | 0.8 | 0.7-1.1 |  | 86/58 | 0.8 | 0.5-1.3 |  | 0.8 | 0.5-1.3 |
| Hydrogen chloride | 352/256 | 62/65 | 0.8 | 0.6-1.1 |  | 0.8 | 0.6-1.1 |  | 10/10 | 0.6 | 0.2-1.6 |  | 0.6 | 0.2-1.8 |
| Soldering fumes | 364/271 | 50/50 | 0.9 | 0.6-1.2 |  | 0.9 | 0.6-1.2 |  | 18/18 | 0.7 | 0.3-1.5 |  | 0.7 | 0.3-1.6 |
| Liquid fuel combustion products | 369/279 | 45/42 | 0.9 | 0.6-1.3 |  | 0.9 | 0.6-1.3 |  | 12/19 | 0.4 | 0.2-1.0 |  | 0.4 | 0.2-1.0 |
| Propane combustion products | 372/288 | 42/33 | 1.3 | 0.8-1.9 |  | 1.2 | 0.8-1.9 |  | 8/8 | 1.1 | 0.3-3.7 |  | 1.1 | 0.3-4.0 |
| Turpentine | 381/301 | 33/20 | 1.2 | 0.8-2.0 |  | 1.3 | 0.8-2.2 |  | 17/11 | 1.1 | 0.4-2.9 |  | 1.3 | 0.5-3.3 |
| Asphalt | 374/277 | 40/44 | 0.7 | 0.5-1.1 |  | 0.7 | 0.4-1.0 |  | 14/16 | 0.4 | 0.2-1.0 |  | 0.4 | 0.2-1.1 |
| Coal tar and pitch | 387/297 | 27/24 | 0.8 | 0.5-1.4 |  | 0.8 | 0.5-1.3 |  | 12/5 | 1.4 | 0.4-5.2 |  | 1.3 | 0.3-4.7 |

* Criteria for selection of chemical agents: at least 10 cases or 10 controls with substantial exposure in the pooled studies; prevalence of exposure greater than 5% among construction workers; and prevalence among controls at least twice as high in construction workers as in other workers.

^†^ Substantial exposure comprised subjects who had been exposed with a probable or definite reliability to medium or high concentrations for more than 5% of their workweek and for at least 5 years.

^‡^ For each chemical agent, the reference category consists of construction workers who were never exposed to the agent.

^§^ Number of cases/number of controls.

^||^ OR_1_ : Odds ratio adjusted for age, median family income for census tract, comprehensive smoking index, respondent status, education level and ethnicity and binary indicator for study.

^¶^ 95% confidence interval.

^**^ OR_2_ : Odds ratio adjusted for same covariates as OR_1_, and binary indicators of exposure for asbestos, crystalline silica and diesel engine emissions.

**Table S5.** Odds ratios of lung cancer and exposure to selected chemical agents in analyses restricted to construction workers, pooled set of studies, using two sets of covariates, and restricted to self-respondents

| Chemical agent* | Never exposed^‡^ | Ever Exposed | | | | | |  | Substantially exposed^†^ | | | | | |
| --- | --- | --- | --- | --- | --- | --- | --- | --- | --- | --- | --- | --- | --- | --- |
|  |  | Ca/Co^§^ | OR_c_^\|\|^ | 95% CI^¶^ |  | OR_a_^**^ | 95% CI |  | Ca/Co | OR_c_ | 95% CI |  | OR_a_ | 95% CI |
| Inorganic insulation dust | 199/184 | 104/104 | 1.0 | 0.7-1.3 |  | 0.9 | 0.7-1.3 |  | 37/32 | 1.1 | 0.6-2.0 |  | 0.9 | 0.5-1.8 |
| Soil dust | 173/145 | 130/143 | 1.0 | 0.8-1.4 |  | 0.9 | 0.6-1.2 |  | 61/28 | 1.8 | 1.0-3.3 |  | 1.4 | 0.7-2.6 |
| Asbestos | 209/200 | 94/88 | 1.2 | 0.9-1.6 |  | 1.1 | 0.8-1.5 |  | 21/12 | 1.8 | 0.8-4.4 |  | 1.7 | 0.7-4.2 |
| Cristalline silica | 115/143 | 188/145 | 1.3 | 0.9-1.7 |  | 1.2 | 0.9-1.6 |  | 58/35 | 1.8 | 1.0-3.2 |  | 1.7 | 0.9-3.1 |
| Portland cement | 202/192 | 101/96 | 1.2 | 0.9-1.6 |  | 1.0 | 0.7-1.4 |  | 42/25 | 1.9 | 1.0-3.6 |  | 1.5 | 0.8-3.0 |
| Glass fibers | 251/249 | 52/39 | 1.1 | 0.8-1.6 |  | 1.1 | 0.8-1.6 |  | 11/10 | 0.9 | 0.3-2.4 |  | 0.8 | 0.3-2.3 |
| Brick dust | 250/238 | 53/50 | 1.1 | 0.8-1.7 |  | 1.0 | 0.7-1.5 |  | 17/7 | 2.0 | 0.7-5.9 |  | 1.7 | 0.6-5.0 |
| Concrete dust | 171/133 | 132/155 | 0.9 | 0.7-1.1 |  | 0.7 | 0.6-1.0 |  | 58/42 | 0.9 | 0.6-1.6 |  | 0.7 | 0.4-1.3 |
| Mineral wool fibers | 218/210 | 85/78 | 1.1 | 0.8-1.5 |  | 1.1 | 0.8-1.5 |  | 20/20 | 1.1 | 0.5-2.5 |  | 1.1 | 0.5-2.4 |
| Calcium oxide | 254/247 | 49/41 | 1.2 | 0.8-1.7 |  | 1.1 | 0.7-1.6 |  | 27/17 | 2.1 | 1.0-4.4 |  | 1.7 | 0.8-3.7 |
| Calcium sulfate | 163/167 | 140/121 | 1.2 | 0.9-1.6 |  | 1.2 | 0.9-1.6 |  | 59/43 | 1.4 | 0.8-2.4 |  | 1.4 | 0.8-2.4 |
| Calcium carbonate | 231/200 | 72/88 | 1.1 | 0.8-1.5 |  | 0.9 | 0.7-1.3 |  | 23/19 | 1.2 | 0.6-2.7 |  | 1.0 | 0.5-2.2 |
| Wood dust | 150/125 | 153/163 | 0.9 | 0.7-1.2 |  | 0.8 | 0.6-1.1 |  | 69/51 | 0.9 | 0.5-1.5 |  | 0.8 | 0.5-1.4 |
| Hydrogen chloride | 252/230 | 51/58 | 0.9 | 0.6-1.2 |  | 0.8 | 0.6-1.2 |  | 8/8 | 0.8 | 0.2-2.4 |  | 0.9 | 0.3-2.8 |
| Soldering fumes | 264/243 | 39/45 | 0.9 | 0.6-1.3 |  | 0.9 | 0.6-1.3 |  | 13/16 | 0.6 | 0.3-1.4 |  | 0.6 | 0.3-1.5 |
| Liquid fuel combustion products | 266/249 | 37/39 | 0.9 | 0.6-1.4 |  | 0.9 | 0.6-1.3 |  | 11/18 | 0.5 | 0.2-1.1 |  | 0.4 | 0.2-1.0 |
| Propane combustion products | 268/256 | 35/32 | 1.2 | 0.8-1.8 |  | 1.1 | 0.7-1.8 |  | 6/7 | 0.8 | 0.2-3.1 |  | 0.9 | 0.2-3.5 |
| Turpentine | 276/268 | 27/20 | 1.2 | 0.7-1.9 |  | 1.3 | 0.8-2.1 |  | 13/11 | 1.0 | 0.4-2.7 |  | 1.2 | 0.4-3.1 |
| Asphalt | 276/244 | 27/44 | 0.6 | 0.4-1.0 |  | 0.6 | 0.4-0.9 |  | 9/16 | 0.4 | 0.1-0.9 |  | 0.3 | 0.1-0.9 |
| Coal tar and pitch | 284/264 | 19/24 | 0.8 | 0.5-1.3 |  | 0.7 | 0.4-1.2 |  | 7/5 | 1.2 | 0.3-4.5 |  | 1.0 | 0.3-3.9 |

* Criteria for selection of chemical agents: at least 5 cases or 5 controls with substantial exposure in the pooled studies; prevalence of exposure greater than 5% among construction workers; and prevalence among controls at least twice as high in construction workers as in other workers.

^†^ Substantial exposure comprised subjects who had been exposed with a probable or definite reliability to medium or high concentrations for more than 5% of their workweek and for at least 5 years.

^‡^ For each chemical agent, the reference category consists of construction workers who were never exposed to the agent.

^§^ Number of cases/number of controls.

^||^ Odds ratio adjusted for age, median family income for census tract, comprehensive smoking index, respondent status, education level and ethnicity and binary indicator for study.

^¶^ 95% confidence interval.

^**^ Odds ratio adjusted for same covariates as previous model, and a binary indicator of exposure for asbestos, crystalline silica and diesel engine emission

**Table S6**. Odds ratios of lung cancer and ever being employed in the construction industry, the reference unexposed category being either all workers outside the construction industry or all blue collar workers outside the construction industry, with or without adjustment for socio-economic status (SES, represented by median family income for census tract and education level).

|  | All workers | | | | | |  | Blue collar workers* | | | | | |
| --- | --- | --- | --- | --- | --- | --- | --- | --- | --- | --- | --- | --- | --- |
|  | Ca/Co^†^  (857/533) | Main results | |  | No SES adjustment | |  | Ca/Co  (703/397) | Main results | |  | No SES adjustment | |
|  |  | OR_1_^‡^ | 95% CI^§^ |  | OR_2_^\|\|^ | 95% CI |  |  | OR_1_^‡^ | 95% CI^§^ |  | OR_2_^\|\|^ | 95% CI |
| Never in the construction industry | 1179/1106 | 1.00 | - |  | 1.00 | - |  | 932/793 | 1.00 | - |  | 1.00 | - |
| Ever in the construction industry | 414/321 | 1.15 | 0.94-1.41 |  | 1.20 | 0.98-1.46 |  | 381/288 | 1.11 | 0.90-1.38 |  | 1.13 | 0.92-1.4 |
| Sector of the construction industry |  |  |  |  |  |  |  |  |  |  |  |  |  |
| Building, industrial, heavy construction^¶^ | 249/195 | 1.26 | 0.98-1.62 |  | 1.34 | 1.05-1.71 |  | 227/170 | 1.23 | 0.94-1.61 |  | 1.28 | 0.98-1.66 |
| Trades contracting^**^ | 202/163 | 1.02 | 0.78-1.33 |  | 1.03 | 0.8-1.34 |  | 187/152 | 0.98 | 0.74-1.29 |  | 0.96 | 0.73-1.26 |
| Duration in the construction industry |  |  |  |  |  |  |  |  |  |  |  |  |  |
| ≥ 10 years | 268/206 | 1.13 | 0.89-1.44 |  | 1.17 | 0.92-1.48 |  | 250/193 | 1.08 | 0.84-1.39 |  | 1.09 | 0.85-1.4 |
| ≥ 20 years | 173/138 | 1.10 | 0.82-1.46 |  | 1.12 | 0.84-1.48 |  | 161/130 | 1.05 | 0.78-1.41 |  | 1.04 | 0.77-1.39 |
| ≥ 30 years | 100/81 | 1.11 | 0.77-1.60 |  | 1.12 | 0.78-1.61 |  | 95/76 | 1.08 | 0.74-1.58 |  | 1.06 | 0.73-1.54 |

^*^ At least 50% of the entire working lifetime spent in blue collar occupations (as defined by Ahrens et al., 1998^40^)

^†^ Number of cases/number of controls.

^‡^ OR_1_: Odds ratio adjusted for age, median family income for census tract, comprehensive smoking index, respondent status, education level and ethnicity.

^§^ 95% confidence interval.

^||^ OR_2_: Odds ratio adjusted for same covariates as OR_1_, except for median family income for census tract and education level.

^¶^ Building, industrial, heavy construction: codes 40 (building, developing and general contracting industries), 41 (industrial and heavy construction industries from the Canadian Standard Industrial Classification of 1980^34^ and codes 404 (building construction), 406 (highway, bridge and street construction) and 409 (other constructions) from the Canadian Standard Industrial Classification of 1970.^33^

^**^ Trade contracting industries: codes 42 (trade contracting industries), 44 (service industries incidental to construction from the Canadian Standard Industrial Classification of 1980^34^ and code 421 (special trade contractors) from the Canadian Standard Industrial Classification of 1970.^33^

**Table S7**: Odds ratios of lung cancer and exposure to selected chemical agents in analyses restricted to construction workers, pooled set of studies, with or without adjustment for socio-economic status (SES, represented by median family income for census tract and education level).

| Chemical agent* | Never exposed^‡^ | Ever Exposed | | | | | |  | Substantially exposed^†^ | | | | | |
| --- | --- | --- | --- | --- | --- | --- | --- | --- | --- | --- | --- | --- | --- | --- |
|  |  | Ca/Co^§^ | OR_1_^\|\|^ | 95% CI^¶^ |  | OR_2_^**^ | 95% CI |  | Ca/Co | OR_1_ | 95% CI |  | OR_2_ | 95% CI |
| Inorganic insulation dust | 272/204 | 142/117 | 0.9 | 0.7-1.2 |  | 0.9 | 0.7-1.2 |  | 50/33 | 1.1 | 0.6-2.0 |  | 1.1 | 0.6-2.0 |
| Soil dust | 237/171 | 177/150 | 1.1 | 0.9-1.5 |  | 1.2 | 0.9-1.5 |  | 76/32 | 1.9 | 1.1-3.4 |  | 2.1 | 1.2-3.6 |
| Asbestos | 292/224 | 122/97 | 1.2 | 0.9-1.5 |  | 1.1 | 0.8-1.5 |  | 25/12 | 1.9 | 0.8-4.6 |  | 1.8 | 0.8-4.1 |
| Crystalline silica | 170/159 | 244/162 | 1.2 | 0.9-1.5 |  | 1.2 | 1.0-1.6 |  | 71/37 | 1.7 | 1.0-3.0 |  | 1.8 | 1.0-3.2 |
| Portland cement | 283/215 | 131/106 | 1.1 | 0.8-1.4 |  | 1.1 | 0.9-1.5 |  | 52/27 | 1.7 | 0.9-3.2 |  | 1.8 | 1.0-3.3 |
| Glass fibers | 346/276 | 68/45 | 1.1 | 0.8-1.5 |  | 1.0 | 0.7-1.4 |  | 14/10 | 1.0 | 0.4-2.8 |  | 1.0 | 0.4-2.7 |
| Brick dust | 347/268 | 67/53 | 1.1 | 0.8-1.6 |  | 1.2 | 0.8-1.6 |  | 18/9 | 1.4 | 0.5-3.6 |  | 1.5 | 0.6-4.0 |
| Concrete dust | 235/155 | 179/166 | 0.9 | 0.7-1.2 |  | 1.0 | 0.7-1.2 |  | 69/48 | 0.9 | 0.5-1.5 |  | 1.0 | 0.6-1.6 |
| Mineral wool fibers | 298/233 | 116/88 | 1.1 | 0.8-1.4 |  | 1.0 | 0.8-1.4 |  | 29/21 | 1.2 | 0.5-2.5 |  | 1.2 | 0.6-2.5 |
| Calcium oxide | 352/277 | 62/44 | 1.1 | 0.8-1.6 |  | 1.1 | 0.8-1.6 |  | 36/18 | 2.0 | 1.0-4.2 |  | 2.0 | 1.0-4.1 |
| Calcium sulfate | 227/187 | 187/134 | 1.2 | 0.9-1.5 |  | 1.1 | 0.9-1.5 |  | 76/44 | 1.5 | 0.9-2.5 |  | 1.3 | 0.8-2.2 |
| Calcium carbonate | 320/230 | 94/91 | 1.0 | 0.8-1.4 |  | 1.1 | 0.8-1.5 |  | 27/19 | 1.2 | 0.6-2.6 |  | 1.3 | 0.6-2.7 |
| Wood dust | 204/141 | 210/180 | 0.9 | 0.7-1.1 |  | 0.9 | 0.7-1.2 |  | 86/58 | 0.8 | 0.5-1.3 |  | 0.9 | 0.5-1.4 |
| Hydrogen chloride | 352/256 | 62/65 | 0.8 | 0.6-1.1 |  | 0.8 | 0.6-1.1 |  | 10/10 | 0.6 | 0.2-1.6 |  | 0.6 | 0.2-1.5 |
| Soldering fumes | 364/271 | 50/50 | 0.9 | 0.6-1.2 |  | 0.8 | 0.6-1.1 |  | 18/18 | 0.7 | 0.3-1.5 |  | 0.6 | 0.3-1.3 |
| Liquid fuel combustion products | 369/279 | 45/42 | 0.9 | 0.6-1.3 |  | 0.9 | 0.6-1.3 |  | 12/19 | 0.4 | 0.2-1.0 |  | 0.4 | 0.2-1.0 |
| Propane combustion products | 372/288 | 42/33 | 1.3 | 0.8-1.9 |  | 1.2 | 0.8-1.9 |  | 8/8 | 1.1 | 0.3-3.7 |  | 1.1 | 0.3-3.5 |
| Turpentine | 381/301 | 33/20 | 1.2 | 0.8-2.0 |  | 1.2 | 0.7-1.9 |  | 17/11 | 1.1 | 0.4-2.9 |  | 1.1 | 0.4-2.8 |
| Asphalt | 374/277 | 40/44 | 0.7 | 0.5-1.1 |  | 0.8 | 0.5-1.1 |  | 14/16 | 0.4 | 0.2-1.0 |  | 0.4 | 0.2-1.0 |
| Coal tar and pitch | 387/297 | 27/24 | 0.8 | 0.5-1.4 |  | 0.9 | 0.5-1.4 |  | 12/5 | 1.4 | 0.4-5.2 |  | 1.3 | 0.4-4.8 |

* Criteria for selection of chemical agents: at least 10 cases or 10 controls with substantial exposure in the pooled studies; prevalence of exposure greater than 5% among construction workers; and prevalence among controls at least twice as high in construction workers as in other workers.

^†^ Substantial exposure comprised subjects who had been exposed with a probable or definite reliability to medium or high concentrations for more than 5% of their workweek and for at least 5 years.

^‡^ For each chemical agent, the reference category consists of construction workers who were never exposed to the agent.

^§^ Number of cases/number of controls.

^||^ OR_1_ : Odds ratio adjusted for age, median family income for census tract, comprehensive smoking index, respondent status, education level and ethnicity and binary indicator for study.

^¶^ 95% confidence interval.

^**^ OR_2_ : Odds ratio adjusted for same covariates as OR_1_, except for median family income for census tract and education level.
